# Contraceptive experience and perception, a survey among Ukrainian women

**DOI:** 10.1186/s12905-018-0651-8

**Published:** 2018-09-29

**Authors:** Volodymyr Podolskyi, Kristina Gemzell-Danielsson, Lena Marions

**Affiliations:** 1 Institute of Pediatrics, Obstetrics and Gynecology of Ukraine, Kiev, Ukraine; 2 Department of Women’s and Children’s Health, Division of Obstetrics and Gynecology, Karolinska Institutet, and Karolinska University Hospital, Stockholm, Sweden; 3 Department of Clinical Science and Education, Södersjukhuset, Karolinska Institutet, Stockholm, Sweden

**Keywords:** Contraception, Survey, Long acting reversible contraception, Ukraine

## Abstract

**Background:**

Abortion rate in Ukraine is high and the use of effective contraceptive methods is low. Aiming to explore women’s knowledge and attitudes towards modern contraceptive methods, we performed a survey among women with a recent pregnancy.

**Methods:**

A convenience sample of 500 women who had an abortion or a delivery (250 women post abortion and 250 women post partum) in Kiev, Ukraine was chosen to participate in the study. A self-administered questionnaire which included questions regarding demographics, plans for future pregnancy, and contraceptive usage, knowledge and the main barriers to contraceptive uptake was distributed.

**Results:**

Most women in our study expressed a wish to postpone or refrain from future pregnancies after the current abortion or delivery. The experience of and the knowledge regarding long acting contraception (LARC) such as intrauterine contraception (IUC) and implants were however low. Barrier methods and oral contraceptives were the most commonly used methods while only a few women had used IUC.

**Conclusion:**

Since most of the respondents did not want a pregnancy in the near future, the findings from this study thus indicate a low uptake for effective and acceptable contraceptive methods and especially LARC methods. Increasing the availability of LARC methods as well as adequate and updated information from providers are essential to reduce the rate of unplanned pregnancy and abortion among Ukrainian women.

## Background

Ukraine was one of the 189 countries that signed United Nations Millennium Declaration [A/RES/55/2] committing to achieve the Millennium Development Goals by 2015. Contraceptive prevalence rate and unmet need for family planning are important indicators of achievement of Target 5.B –Universal access to reproductive health. Recent studies show worldwide increasing contraceptive prevalence (from 54.8% in 1990 to 63.3% in 2010) and a decrease in unmet need for family planning (from 15,4% in 1990 to 12,3% in 2010), in Ukraine there was no rapid change in contraceptive prevalence rate (from 66,6% in 1990 to 67,0% in 2010) while the unmet need for family planning decreased from 11,6% in 1990 to 10,7% in 2010 [1].

The total unmet need for family planning is calculated as the number of women of reproductive age who are heterosexually active, with no current desire of childbearing and are not using a contraceptive method.

Contraceptive use among married women 15–49 years of age in Ukraine slightly decreased from 67,5% in 1999 to 65,4% in 2012, while the barrier method use increased from 13,5% to 24,2% during this period according to official statistics. [2]. Since the unmet need for family planning is the gap between women’s reproductive intentions and their contraceptive behavior it is important to reveal contraceptive prevalence, which together with unmet need for family planning identifies total demand for family planning [3]. The population of Ukraine was growing during the first five years after independence until it reached 52,244,000 in 1993, gradually decreased until 1990 and again increased to the current average population of 42,800,501 in 2013. According to official statistics, total fertility rate (TFR) in Ukraine in 2013 was 1.5 per woman aged 15–49 years, it has been decreased from 1.77 in the 1991, when the country became independent [4]. Age-specific fertility rates in 2012 appeared to be highest in the age group of 20–24 [5].

Counseling for family planning methods are usually performed by obstetricians and gynecologists either in family planning clinics, out-patient clinics or in hospitals (maternity houses).

The access to contraceptive methods depends on the knowledge among health care providers (HCPs) and on the income, per capita in the region. Among long acting reversible contraceptive (LARC) methods only intrauterine contraceptives (IUC) are available in the country. Implants are still, as of August 2017, not available in Ukraine. The cost of copper- intrauterine device (IUD) is around 15–20 EUR and for the levonorgestrel releasing intrauterine system (LNG-IUS) approximately 700 EUR. Injectable contraceptives which contain medroxyprogesterone acetate cost 15–20 EUR per injection and the cost for oral contraceptive pills is approximately 10–15 EUR for 3 months’ supply. The economical crisis in Ukraine during 2014–2015 contributed to an abrupt increase of cost for imported drugs, which also affected hormonal contraceptives. Advertising for modern LARC methods at Ukrainian social media is not permitted, however there are many other forums and websites, were women and HCPs are sharing the experience of the use of hormonal contraception.

Abortion on request is permitted in Ukraine until the 12th week of gestation. The most common method is dilatation and curettage (D&C) which is performed in both governmental and private clinics. Healthcare is free for Ukrainian citizens, per the constitution, making the abortion procedure free, however if need for additional treatment or in case of complications, such as infection or prolonged bleeding, women must pay everything themselves. Medical abortion, using mifepristone and misoprostol, is also available in Ukraine, but it is expensive and all medication used is paid by the woman.

In 1995 the abortion rate in Ukraine was among the highest in Europe – 58,2 per 1000 women aged 15–49 years and higher than the average worldwide abortion rate (35 per 1000 women aged 15–49 years). According to official abortion statistics abortion rates rapidly declined during 1995–2005 and after that the drop notably slowed (from 41,3% in 1995–2000 to 9,3% in 2009–2010). [2] The birth rate in Ukraine in 2016 was 10.5 births per 1000 population. [6]

With the objective to obtain a view of the current situation regarding family planning methods in Ukraine we performed the present study aiming to explore the current experience and knowledge regarding contraceptive methods among recently pregnant women and to identify possible barriers for the use of effective methods such as LARC methods.

## Methods

A survey was carried out among women attending two abortion clinics and three gynecological units in Kiev, Ukraine. The gynecological units were located at two Public Hospitals and in the postpartum clinic of the Institute of Pediatrics, Obstetrics and Gynecology of National Academy of Medical Sciences of Ukraine in Kiev.

A convenience sample of 500 women who had an abortion or a delivery (250 women post abortion and 250 women post partum) were consecutively invited to participate in the study. Before women were discharged from the clinic, study related information was provided by the doctor in charge, and women signed informed consent prior to participation in the study.

A self-administered questionnaire was used which included questions regarding demographics (age, parity, gravidity, relationship status and educational level), plans for future pregnancy, and 11 open ended questions regarding contraceptive usage, knowledge and source of information as well as main barriers to contraceptive uptake. The question regarding contraceptive experience was “what contraceptive method have you ever used”, and for knowledge the question was “what contraceptive method have you heard about”. More than one alternative could be chosen. The list of possible methods consisted of Contraceptive pill, Progestogen-only contraceptive pill, Contraceptive implant, Contraceptive ring or patch, Contraceptive injection, IUD with copper, IUD with hormone, Condoms, Diaphragm, Rhythm, Withdrawal, None. Women completed the questionnaire during their stay at the clinic. A pilot survey of 50 women was performed prior to the main study. Questionnaires were answered either by women in their single rooms or in a separate private room at the postpartum unit. Women who had undergone an abortion filled out the questionnaire in a separate room at the gynecological unit or in the abortion clinic. The questionnaires were distributed from January to August 2015. During the study period, there were 324 women attending the abortion clinic and 282 women that had a delivery at the maternity clinic and 83% (500/606) were included in the study.

The aim of the study was to explore contraceptive uptake and knowledge among recently pregnant women (post abortion and post partum women). The main outcome was the contraceptive experience among these women. This was assessed as the number of women who had ever used contraceptive methods. Secondary outcomes were contraceptive knowledge and sources of knowledge, plans for future pregnancies and barriers for contraceptive use such as perceived or feared side effects.

Ethical permission for the study was received from the ethics committee of the Institute of Pediatrics, Obstetrics and Gynecology of the National Academy of Medical Sciences of Ukraine (7/2013-12-27).

## Results

Most women included in the survey had a current partner. There were more women in the postpartum group that had one or two previous pregnancies compared to women in the post abortion group (58% and 50% respectively). (Table 1).

**Table 1:** Demographic characteristics of surveyed postabortion and postpartum women in Ukraine

Age(years)	Postabortion group (*n* = 250)	Postpartum group (n = 250)
	n (%)	n (%)
Mean age	29.6 (+/−3.7)	27.2 (+/−3.1)
15–19		4 (2)
20–25	44 (18)	47 (19)
26–30	75 (30)	87 (35)
31–35	60 (24)	62 (25)
36–40	46 (18)	20 (8)
41–45	13 (5)	5 (2)
46–49	5 (2)	1 (0,4)
Missing data	7 (3)	24 (10)
Relationship status		
Current partner	196 (78)	221 (88)
No partner	33 (13)	9 (4)
Missing data	21 (8)	20 (8)
Number of previous pregnancies		
0	56 (22)	72 (29)
1–2	126 (50)	146 (58)
≥ 3	68 (27)	32 (13)
Number of previous deliveries		
0	75 (30)	82 (33)
1–2	167 (67)	157 (63)
≥ 3	8 (3)	11 (4)
Education level		
Secondary education incomplete (Middle school)	4 (2)	4 (2)
Secondary education completed (High school)	61 (24)	44 (18)
College	56 (22)	36 (14)
University	129 (52)	166 (66)

**Fig. 1:**
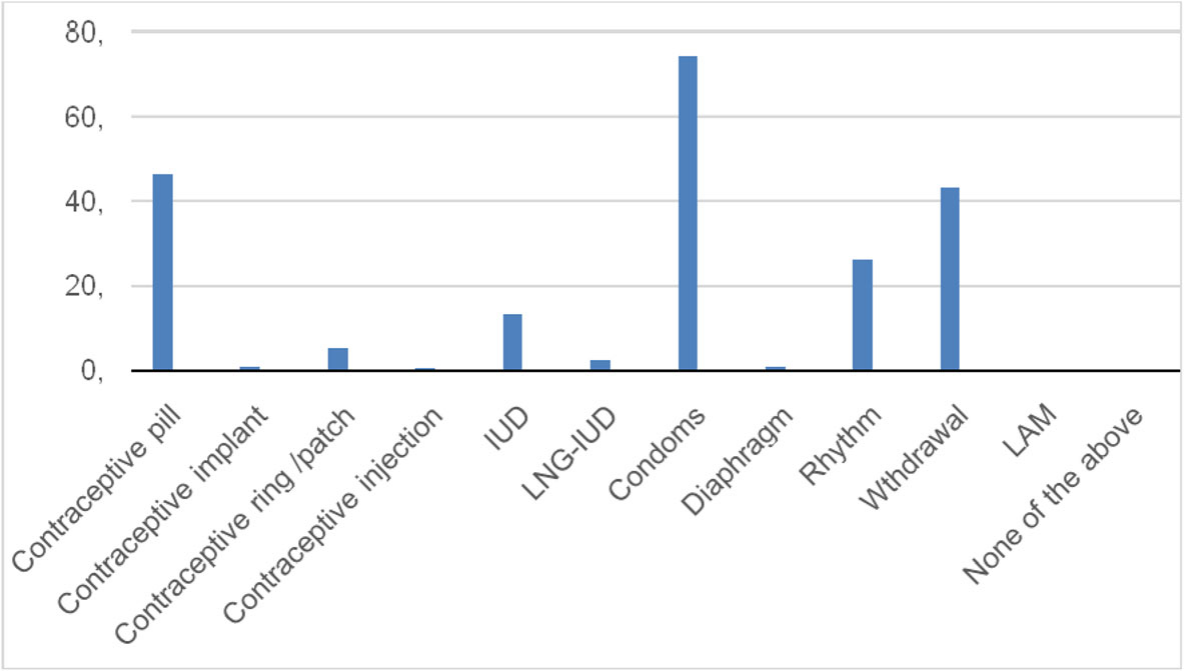
Contraceptive experiences among postabortion and postpartum women in Ukraine

**Fig. 2:**
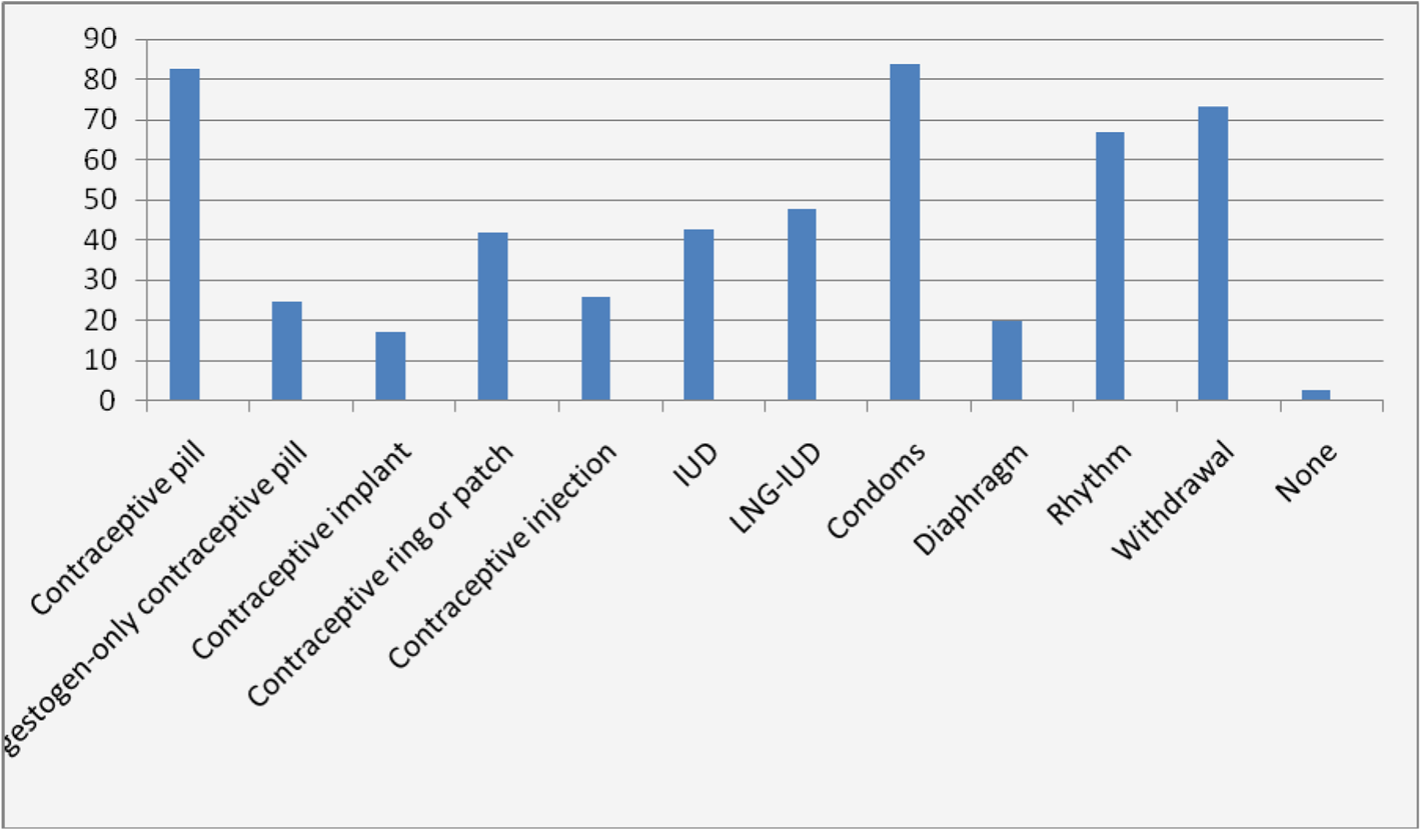
Contraceptive knowledge among postabortion and postpartum women in Ukraine

Barrier methods (condoms and pessaries) and oral contraceptives were the most commonly used methods (75% and 46% respectively) whilst only a few women had used IUC (16%). (Fig. 1) The knowledge regarding LARC methods such as IUC and implants were low (17% and 47% respectively). (Fig. 2).

Most women (89%) stated that information regarding contraceptive methods was obtained from a medical doctor. Other sources of information were internet and friends. Most women (82%) also expressed a view that a medical doctor constituted their preferred source of information.

More than a half of surveyed women, (67%) planned to wait at least 3 years before having a child or to refrain from further pregnancies. Thirty-seven women (9%) expressed a wish for a future pregnancy within one year and 24% wanted to wait between one and three years.

Most common self-reported side effects from hormonal contraceptive methods reported by women in the study were weight gain (21%), mood changes (15%), headache (14%) and breast tenderness (10%), while 50% of all women had not reported any side effects (Table 2).

**Table 2:** Ever experienced self-reported side effects of hormonal contraception among postabortion and postpartum women in Ukraine

*n* = 500	All women n (%)
Acne	22 (4)
Pelvic pain	32 (6)
Loss of libido	47 (9)
Increased discharge	33 (7)
Headache	98 (20)
Thrombosis	8 (2)
Bloating	33 (7)
Weight gain	107 (21)
Skin discolouration (pigmentation)	8 (2)
Irregular bleeding	43 (9)
Mood changes	75 (15)
Breast tenderness	49 (10)
None	250 (50)

The most feared self-reported side effects related to hormonal contraception were future infertility expressed by 177 women (35%), followed by thrombosis expressed by 118 (24%) and 107 women (21%) feared weight gain.

## Discussion

We found that the experience of - as well as knowledge on - effective contraceptive methods were low among the recently pregnant women assessed. Most women were using perceived natural methods (withdrawal or rhythm methods), condoms or oral contraception. The use of hormonal methods was also limited by reported or feared side-effects. Many of these self-reported side-effects are not causally related to hormonal contraception but constitute a barrier for using them. This is in line with a report from Sweden where fear of side-effects, was found to be a common reason for discontinuation of hormonal contraception [7]. Very few women had knowledge of or experience from the use of LARC-methods. This finding in combination with the fact that more than half of all women expressed a wish to refrain from future pregnancies or postpone childbearing for at least three years indicate low contraceptive uptake among these women. Intrauterine contraception and subdermal implants constitute the most effective contraceptive methods especially for women opting for long-acting methods. Our study confirms previous findings [8] that Ukrainian women prefer natural methods, condoms or oral contraception. This was also the finding in a recent study among Romanian female university students [9]. This is however in contrast with a national Multiple Indicator Cluster Survey in Ukraine for 2005 [5] that reported IUC to be the most commonly used method for contraception. The discrepancy between these findings might reflect geographical differences. High rates of IUC usage was found in a study performed in the Western region of Ukraine in 2003 [10] while reports from other regions during the same period show barrier methods as well as induced abortion as the preferred methods for family planning [11, 12]. The possible reason for this difference might reflect the fact that the Western region is closely bordering two European countries making it possible for women [13], to access information on modern contraceptive methods from advertisement in the neighboring countries [14]. Such information is not available for all women in Ukraine at present. Neither is the information available for providers which of course are crucial to be able to increase the uptake and change the attitudes regarding effective methods for family planning. Data from Russia indicate a decline in the use of the most effective contraceptive methods such as IUC due to provider attitudes, probably by affecting women’s fear and knowledge [15].

High rates of barrier methods such as condoms have also previously been shown among Ukrainian women, likely due to a nationwide AIDS information campaign [11]. Since 1987 Ukraine was experiencing extremely high rates of HIV among drug users (60% of all reported cases from 1987 to 2004) and efforts of Ministry of Health of Ukraine resulted in the development of the Nationwide AIDS Program held in periods starting in 2001 [16]. While condom use is the only method for prevention of sexually transmitted infections, its efficacy for prevention of unplanned pregnancy is less than hormonal methods and much lower than for LARC methods such as implants or IUCs [14, 17]. However these latter methods are not yet available for all women in Ukraine.

Strengths and weaknesses. The strength of the study is that women included in the survey were recently pregnant women in the age groups that are in most need for effective family planning methods. The result from our study also confirms that many women still have limited knowledge and express misperception and fear towards modern contraceptive methods. Women in our study stated that medical doctors constituted their main source of information and unfortunately, we did not explore the knowledge regarding contraceptive methods among medical doctors and other contraception providers. This gap of knowledge needs to be addressed in future studies.

Another limitation is the small sample size, which makes it difficult to generalize the findings to all women in the country.

## Conclusions

Most women in our study wanted to postpone or refrain from future pregnancies after abortion or delivery but had very limited experience and knowledge regarding effective contraceptive methods. The findings from this study thus indicate low contraceptive uptake of effective contraceptive methods and especially LARC methods. Increasing the availability of LARC methods as well as ensuring adequate and updated information from health care providers are essential steps to reduce the rate of unplanned pregnancy and abortion among Ukrainian women. However, a successful intervention needs to focus also on education for gynecologists and other providers of contraception. Information to women must include relevant facts regarding advantages and disadvantages with hormonal methods. We found that many women believed in old myths regarding hormonal contraception such as increased risk for future infertility. There is a need for policy makers in collaboration with professional associations in the country to promote different activities aiming to increase the uptake of effective contraceptive methods. Education for teachers, general awareness and availability of affordable methods are important. Education in sexual and reproductive health, for young girls as well as for young boys, is also essential and must begin early in life.

## Abbreviations

HIV: human immunodeficiency virus
IUC: intrauterine contraception
IUD: intrauterine device
LARC: long acting reversible contraception
TFR: total fertility rate

## References

[1] Alkema Leontine, Kantorova Vladimira, Menozzi Clare, Biddlecom Ann (2013). National, regional, and global rates and trends in contraceptive prevalence and unmet need for family planning between 1990 and 2015: a systematic and comprehensive analysis. The Lancet.

[2] State Statistics Service of Ukraine (2014a). Demographic data for 2013. http://www.ukrstat.gov.ua/operativ/operativ2007/ds/nas_rik/nas_e/nas_rik_e.html. Accessed 23 Nov 2015.

[3] The updated handbook on Indicators for Monitoring the Millennium Development Goals (http://mdgs.un.org/unsd/mi/wiki/5-6-Unmet-need-for-family-planning.ashx).

[4] https://data.worldbank.org/indicator/SP.DYN.TFRT.IN?. Accessed 10 Aug 2017.

[5] Ukraine - Multiple Indicator Cluster Survey 2012 United Nations Children’s Fund State Statistics Service of Ukraine Ukrainian Institute for Social Reforms Statinformconsulting. (https://www.unicef.org/ukraine/Ukraine_MICS_FinalReport_ENG.pdf ). Accessed 23 Nov 2015.

[6] Sedgh, Gilda ; Singh, Susheela ; Shah, Iqbal H ; Åhman, Elisabeth ; Henshaw, Stanley K ; Bankole, Akinrinola Induced abortion: incidence and trends worldwide from 1995 to 2008 Lancet, 18–24 February 2012, Vol.379(9816), pp.625–632. 10.1016/S0140-6736(11)61786-8 22264435

[7] Lindh I, Blohm F, Andersson-Ellström A, Milsom I (2009). Contraceptive use and pregnancy outcome in three generations of Swedish female teenagers from the same urban population. Contraception.

[8] Ukraine 2007: results from the Demographic and Health Survey. Stud Fam Plann. 2010 Sep;41(3). 10.1111/j.1728-4465.2010.00246.x 21469274

[9] Blidaru Iolanda Elena, Furau Gheorghe, Socolov Demetra (2015). Female Romanian university students’ attitudes and perceptions about contraception and motherhood. The European Journal of Contraception & Reproductive Health Care.

[10] Muscato L., Kidd R. S. (2003). Contraception and abortion attitudes and practices of Western Ukraine women. The European Journal of Contraception & Reproductive Health Care.

[11] Mogilevkina I., Odlind V. (2003). Contraceptive practices and intentions of Ukrainian women. The European Journal of Contraception & Reproductive Health Care.

[12] Mogilevkina Iryna, Tydén Tanja, Odlind Viveca (2001). Ukrainian Medical Students' Experiences, Attitudes, and Knowledge About Reproductive Health. Journal of American College Health.

[13] Report from the Commission to the European Parliament and the Council on the implementation and functioning of the local border traffic regime introduced by Regulation (EC) No 1931/2006 of the European Parliament and of the Council laying down rules on local border traffic at the external land borders of the Member States /* COM/2009/0383 final */. (http://eur-lex.europa.eu/legal-content/EN/TXT/?uri=celex:52009DC0383). Accessed 3 Dec 2015.

[14] Buhling Kai J., Hauck Brian, Dermout Sylvia, Ardaens Katty, Marions Lena (2014). Understanding the barriers and myths limiting the use of intrauterine contraception in nulliparous women: results of a survey of European/Canadian healthcare providers. European Journal of Obstetrics & Gynecology and Reproductive Biology.

[15] Perlman F, McKee M (2009). Trends in family planning in Russia, 1994-2003. Perspect Sex Reprod Health.

[16] Johnson, N . Crossing the border for the pill: an analysis of the decision to purchase Oral contraceptives over-the-counter from Mexican pharmacies. MPA/MPP capstone projects. Paper 9. Lexington, KY: University of Kentucky; 2014. (https://uknowledge.uky.edu/cgi/viewcontent.cgi?referer=&httpsredir=1&article=1008&context=mpampp_etds). Accessed 3 Dec 2015.

[17] Hatcher R.; Trussell J.; Nelson A., eds. (2011). Contraceptive technology (20th ed.). New York: Ardent Media. ISBN 978-1-59708-004-0.

